# Resonant Interlayer Coupling in NbSe_2_‐Graphite Epitaxial Moiré Superlattices

**DOI:** 10.1002/adma.202511262

**Published:** 2025-12-19

**Authors:** Shu Mo, Ksenija Kovalenka, Sebastian Buchberger, Bruno K. Saika, Anugrah Azhar, Akhil Rajan, Andela Zivanovic, Yu‐Chi Yao, Rodion V. Belosludov, Matthew D. Watson, M Saeed Bahramy, Phil D.C. King

**Affiliations:** ^1^ SUPA, School of Physics and Astronomy University of St Andrews St Andrews KY16 9SS UK; ^2^ Department of Physics and Astronomy University of Manchester Manchester M13 9PL UK; ^3^ Max Planck Institute for Chemical Physics of Solids Nöthnitzer Strasse 40 01187 Dresden Germany; ^4^ Physics Study Program, Faculty of Science and Technology Syarif Hidayatullah State Islamic University Jakarta, Tangerang Selatan 15412 Indonesia; ^5^ Institute for Materials Research Tohoku University Sendai 980‐08577 Japan; ^6^ Diamond Light Source Ltd Harwell Science and Innovation Campus Didcot OX11 0DE UK

**Keywords:** 2D materials, electronic structure, graphite, moiré, NbSe_2_

## Abstract

Moiré heterostructures, created by stacking 2D materials together with a finite lattice mismatch or rotational twist, represent a new frontier of designer quantum materials. Typically, however, this requires the painstaking manual assembly of heterostructures formed from exfoliated materials. Here, clear spectroscopic signatures of moiré lattice formation in epitaxial heterostructures of monolayer (ML) NbSe_2_ grown on graphite substrates are observed. Angle‐resolved photoemission measurements and theoretical calculations of the resulting electronic structure reveal moiré replicas of the graphite π states forming pairs of interlocking Dirac cones. Interestingly, these intersect the NbSe_2_ Fermi surface at the k‐space locations where NbSe_2_'s charge‐density wave (CDW) gap is maximal in the bulk. This provides a natural route to understand the lack of CDW enhancement for ML‐NbSe_2_/graphene as compared to a more than fourfold enhancement for NbSe_2_ on insulating support substrates, and opens new prospects for using moiré engineering for controlling the collective states of 2D materials.

## Graphical abstract

Combining angle‐resolved photoemission spectroscopy and density functional theory derived calculations, this work reveals strong interlayer coupling and moiré replicas of the graphite π states in molecular‐beam epitaxy grown van der Waals heterostructures of monolayer‐NbSe2/graphite, with implications for its collective phases that form.

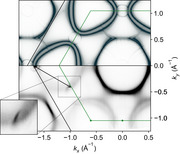

## 1 Introduction

Super‐periodic moiré potentials in 2D materials have been shown to underpin a plethora of highly tunable interacting electronic states,^[^ ^1^, ^2^, ^3^, ^4^ ^]^ including unconventional superconductors,^[^ ^5^, ^6^, ^7^ ^]^ correlated insulators,^[^ ^8^, ^9^, ^10^ ^]^ and Wigner crystals.^[^ ^11^, ^12^, ^13^ ^]^ Almost all studies of the electronic structure of such moiré heterostructures to date, however, have been performed using exfoliated materials.^[^ ^14^, ^15^, ^16^, ^17^ ^]^ While these have been instrumental in helping establish the general phenomenology of 2D moiré systems, significant materials challenges remain,^[^ ^18^ ^]^ including the achievable levels of twist‐angle and strain homogeneity^[^ ^19^, ^20^ ^]^ and the introduction of disorder due to contamination effects induced during the fabrication process.^[^ ^18^, ^21^ ^]^ Despite several pioneering efforts to realise larger‐area van der Waals moiré materials,^[^ ^22^, ^23^ ^]^ this materials complexity has effectively limited the available systems to study to the most stable and air‐insensitive examples of graphene and the semiconducting transition‐metal dichalcogenides (TMDs). Additional approaches for fabricating and controlling moiré materials are thus strongly desired.

In this respect, ultra‐high‐vacuum‐based epitaxial growth techniques should offer an attractive alternative approach to the formation of large‐area and ultra‐clean moiré superstructures from constituent layers hosting different lattice constants. The growth of 2D materials by such methods, however, has traditionally been limited by materials quality, with significant rotational disorder, the formation of small disconnected grains, and premature bilayer formation typically observed.^[^ ^24^, ^25^, ^26^, ^27^ ^]^ Here, motivated by significant recent enhancements in the quality and uniformity of the 2D layers that can be fabricated^[^ ^28^, ^29^ ^]^ and using very slow growth rates to facilitate fabrication of high quality well‐ordered layers (see “Experimental Section”), we explore the prospect of creating TMD moiré heterostructures using molecular‐beam epitaxy (MBE). To this end, we synthesise homogeneous ML‐NbSe_2_/graphite van der Waals heterostructures, achieving an improved crystalline and rotational order and electronic linewidths as compared to previous studies.^[^ ^30^, ^31^, ^32^, ^33^ ^]^ This, in turn, allows us to investigate moiré coupling in this system, which forms between two distinct subsystems hosting markedly different electronic structures. We study the resulting heterostructure using angle‐resolved photoemission spectroscopy (ARPES) and model calculations derived from density functional theory (DFT). Our measurements reveal clear signatures of a well‐defined moiré superstructure being formed, with significant interactions evident between the NbSe_2_ and graphite layers. Our calculations allow us to identify the origin of the strong interlayer interactions as being due to a resonant coupling between the Fermi surfaces of the graphite and NbSe_2_ states, and reveal how this can in turn act in competition with the intrinsic charge density wave order of the NbSe_2_ layer. This opens a new perspective for controlling pre‐existing collective instabilities using moiré potentials, while our all‐epitaxial approach provides a scaleable platform for the creation of large‐area moiré materials.

## 2 Results and Discussion

### 2.1 Electronic Structure of NbSe_2_/Graphite Epitaxial Heterostructures

**Figure 1:**
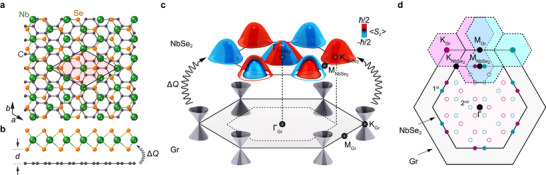
NbSe_2_/graphite van der Waals heterostructures. a,b) Top (a) and side (b) views of the crystal structure of the ML‐NbSe_2_/graphite heterostructures investigated here. A putative interaction between the NbSe_2_ and the topmost graphite layer, leading to an interlayer charge transfer, Δ*Q* is shown. The near coincidence of two unit cells of the NbSe_2_ to three of the graphite surface layer is indicated by the unit cell shown in (a). c) Schematic model of the near‐*E*_F_ electronic structure of the top‐layer graphite and NbSe_2_ layers. d) Brillouin zones of the constituent layers, showing the expected location of first‐order (solid points) and second‐order (open points) moiré replicas of the K (magenta) and K′ (cyan) graphite states, respectively.

**Figure** **1** shows a schematic overview of the NbSe_2_/graphite heterostructures investigated here. Both NbSe_2_ and graphite are van der Waals materials, enabling the formation of high‐quality epitaxial interfaces between the two (Figure 1a,b; see also Figure S1, Supporting Information), despite their large lattice constant mismatch of ≈40%. Unlike most other moiré systems investigated to date, ML‐NbSe_2_ is itself a correlated material, of strong interest for its CDW state^[^ ^30^, ^34^ ^]^ and as a host of Ising superconductivity.^[^ ^35^ ^]^ The latter arises due to the broken inversion symmetry of the monolayer, combined with an inherently strong spin–orbit coupling. Together, this leads to a spin‐polarized electronic structure which is characterized by a locking of the quasiparticle spin to the valley pseudospin^[^ ^35^, ^36^ ^]^ (Figure 1c), much like in the famous 2D semiconductors WSe_2_ and MoS_2_.^[^ ^37^, ^38^, ^39^, ^40^ ^]^ In fact, NbSe_2_ can be considered as a significantly hole‐doped analogue of MoS_2_, with a nominal *d* ^1^ electronic configuration leading to large spin‐valley‐coupled Fermi surfaces located around the Brillouin zone corners (Figure 1c). Although the Brillouin zone of NbSe_2_ is much smaller than that of graphite (Figure 1d), for azimuthally‐aligned layers as shown in Figure 1a, its large Fermi surfaces can be expected to overlap the Dirac states at the K points of the underlying graphite layer. This opens a potential electronic coupling channel between the two layers (Figure 1b,c). If realized, this would raise the exciting prospect to stabilize a rather short‐wavelength moiré heterointerface (Figure 1d) between the strongly lattice‐mismatched NbSe_2_ and graphite layers here; a prospect which we explore in detail below.

**Figure 2:**
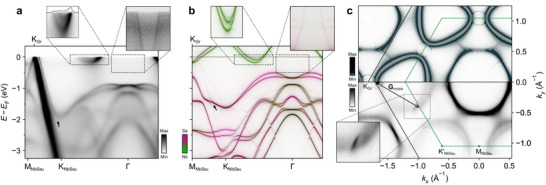
Measured and calculated electronic structure. a) Measured dispersion of the NbSe_2_/graphite heterostructures along the Γ‐K‐M direction, obtained by summing dispersions acquired at *h*ν = 70 eV using circular‐left (CL) and circular‐right (CR) polarized light. A magnified view of the Nb *d*‐orbital metallic bands is shown in the left inset (measured at *h*ν = 55 eV using CL polarization; the black curve is a momentum distribution curve at the Fermi level). The right inset shows a measurement (*h*ν = 55 eV, linear‐vertical polarization) close to Γ with enhanced contrast, showing first‐order Nb and second‐order graphite moiré replicas. b) Corresponding model calculations of the expected dispersion of our heterostructures. The colour shows the atomic character, and the intensity the spectral weight. The inset close to Γ is shown with enhanced contrast. c) Equivalent model calculations (top) and measurements (bottom, *h*ν = 70 eV, CL and CR polarizations) of the Fermi surface. A magnified view of the observed graphite replica is shown in the inset with higher contrast.

The electronic structure as measured from our fabricated epitaxial heterostructure (see “Experimental Section”) is shown in **Figure** **2**a. We resolve a pair of states crossing the Fermi level along the Γ‐K direction (see left inset). These are nearly degenerate close to Γ, but become split by around 150 meV where they cross the Fermi level closer to the zone‐corner K point. We assign these as the Nb 4*d* states, whose band dispersion is in good agreement with our theoretical calculations (Figure 2b, left inset). Consistent with previous studies,^[^ ^35^, ^36^, ^41^ ^]^ our calculations (see “Computational Section”) indicate that the splitting evident in these states derives from the spin–orbit interaction (see Figure S3, Supporting Information), leading to the spin‐valley‐locked texture shown schematically in Figure 1c. This spin splitting has been challenging to observe in electronic structure measurements of single‐layer NbSe_2_ to date.^[^ ^32^, ^42^ ^]^ Here, however, we clearly resolve the band splitting both in our measured dispersions (inset of Figure 2a), and in Fermi surface measurements (bottom panel of Figure 2c), where they contribute a pair of split‐off Fermi pockets around each zone‐corner K and K′ point of the NbSe_2_ Brillouin zone. Their clear resolution here confirms the significant spin–orbit splitting which underpins Ising superconductivity in this system.^[^ ^35^, ^41^ ^]^ Closer to Γ, the spin splitting is predicted to become very small, with spin degeneracies formed along the Γ‐M direction (Figures 1c and 2c). Consistent with this, we resolve only a single hexagonally‐warped Fermi pocket centred at the Brillouin zone centre.

The sharp linewidths and clear momentum‐dependent anisotropy of the Fermi surfaces we observe point to a high sample quality with good quasiparticle lifetimes, as well as excellent rotational order in our fabricated samples. We observe an excellent alignment between the NbSe_2_ and graphite‐derived states, indicating growth of NbSe_2_ that is azimuthally aligned to the underlying graphite lattice, with a negligible twist angle between the layers. Probing strongly anisotropic energy contours at higher binding energies, where we observe dispersive states derived from the Se 4*p* orbitals (Figure 2a,b),^[^ ^31^ ^]^ we can place an upper limit on the twist angle disorder in our samples of ≈1° (see Figure S2, Supporting Information), pointing to the strong potential of MBE here for the fabrication of high‐quality van der Waals heterostructures. Interestingly, where the Se 4*p*‐derived states approach the π states of the graphite layer close to the boundary of the NbSe_2_ Brillouin zone, we find these hybridise (arrow in Figure 2a). This points to a non‐negligible interlayer coupling in this system. Consistent with this, we find that the π states themselves are split, as compared to measurements of the same bands in a pristine graphite sample (see Figure S4, Supporting Information): not only do we observe the expected^[^ ^43^ ^]^ rather broad (due to $k_{z}$‐dispersion) linearly‐dispersing band with its charge neutrality point close to the Fermi level, but also a sharp copy that is shifted to lower binding energy (Figure 2a). The latter contributes a well‐defined hole‐like Fermi pocket at the zone‐corner K point of the graphite Brillouin zone (Figure 2c). It is this latter band that is hybridized with the Se *p*‐states from the NbSe_2_ layer. We thus conclude that the interaction between the NbSe_2_ layer and the graphite beneath is rather strong, with a significant charge transfer from the topmost graphite layer to the NbSe_2_ monolayer (Figure 1b) causing the graphite π states to become energetically split‐off from the bulk‐like graphite states below. The fact that we only measure a single split‐off band strongly suggests that the charge transfer occurs only into the topmost graphite layer, with the deeper layers remaining bulk‐like. This is likely due to the strong screening of the bulk graphite, effectively limiting the charge transfer to the surface‐most layer. From a Luttinger analysis (see Figure S4, Supporting Information), we estimate that the charge transfer causes a substantial hole doping of the topmost graphite layer of ≈2.5 × 10^13^ holes/cm^2^.

### 2.2 Moiré Replica Formation

**Figure 3:**
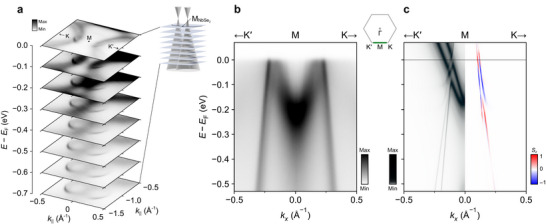
Moiré replicas of the graphite π states. a) Measured constant energy contours (*h*ν = 70 eV, sum of spectra acquired using CL and CR polarizations) in the vicinity of the M point of the NbSe_2_ Brillouin zone. Clear graphite replicas are visible contributing small pockets at the Fermi level, which grow in area and start overlapping with increasing binding energy below *E*_F_, as shown schematically in the inset. b) Measured dispersion (*h*ν = 55 eV, CL and CR polarizations) along the K′‐M‐K direction (as indicated in the inset), showing the sharp graphite replica states. c) Corresponding calculated dispersion along K′‐M‐K, from our supercell calculations. The left‐hand side shows the calculations projected onto the NbSe_2_ layer, while the right shows the spin polarization of the graphite states when projected onto the C orbitals.

Besides this charge transfer and signatures of interlayer hybridization, the electronic structure features described above would be broadly expected from considering that of individual NbSe_2_ and graphite layers. We find, however, that there are additional features in the measured electronic structure that can only be obtained for the heterostructure configuration. Most obviously, we observe weak but sharp arc‐like features in our measured Fermi surface (Figure 2c; see also Figures S5 and S7, Supporting Information), intersecting each of the Nb‐derived zone‐corner Fermi pockets close to the M point of the NbSe_2_ Brillouin zone. We show a magnified view of one of these in the inset of Figure 2c, and constant energy contours in the vicinity of K‐M‐K′ in **Figure** **3**a. From these, it is evident that the observed arcs are in fact one side of a closed trigonally‐warped pocket. A clear linear dispersion is evident in our measurements of these features performed along the K‐M‐K′ direction of the NbSe_2_ Brillouin zone (Figure 3b), with two cone‐like features visible in our constant energy surfaces. These cones start to intersect with increasing energy below the Fermi level. The size and shape of the resulting pockets are, in fact, in excellent agreement with those of the sharp π state of the topmost graphite layer (Figure 2c; Figure S5, Supporting Information), but shifted in momentum by the moiré vector of ≈0.85 Å^−1^ defined by the lattice mismatch of the NbSe_2_ and graphite layers (Figure 1d). We thus attribute the additional sharp features observed here as moiré replicas of the graphite states^[^ ^44^ ^]^ coming from neighbouring K and K′ points of the original graphite Brillouin zone.

To confirm this assignment, we turn to our DFT‐derived model calculations, which explicitly include an interlayer coupling between the NbSe_2_ and a single graphite layer. We consider a commensurate lattice match between two unit cells of the NbSe_2_ and three of the graphite. This provides a computationally‐tractable system size which is nonetheless quite close to the experimental situation (see “Computational Section”). Our calculations in Figures 2b,c and 3c, which are projected onto the orbitals of the NbSe_2_ layer, show excellent agreement with our experimental measurements. In particular, close to the M point of the NbSe_2_ Brillouin zone, two trigonally‐warped pockets are evident intersecting the Nb‐derived Fermi pockets (top panel of Figure 2c). These are derived from replicas of the K and K′‐valley graphite states, respectively. They are centred exactly at the M point in our calculations due to the enforced commensuration, while they are slightly offset from this in our experiments due to deviations in the real heterostructure from the assumed 2:3 lattice mismatch. Experimentally, it should be possible to tune between these scenarios by varying the lattice mismatch between the substrate and the epilayer, for example by replacing some Se with Te. This will provide a direct tuning of the moiré wavelength, while the large remaining lattice mismatch should act to overcome lock‐in effects that are known to occur in systems with small lattice mismatches or relative twists.

From the comparison with our model calculations, we therefore deduce that the appearance of the graphene‐like Dirac cone replicas intersecting the NbSe_2_ K‐barrel Fermi surfaces in our experimental measurements constitute a clear spectroscopic signature of moiré‐assisted interlayer tunnelling. These features arise from momentum‐conserving scattering processes in which the mismatch between the reciprocal lattice vectors of the two constituent layers is compensated by the moiré superlattice. This mechanism is analogous to the generalized Umklapp scattering^[^ ^45^, ^46^, ^47^ ^]^ encountered in twisted bilayer graphene, wherein interlayer tunnelling $t(k_{{\text{NbSe}}_{2}},k_{\text{Gr}})$ is governed by the selection rule,

(1) $$\delta (k_{\text{Gr}}+G_{\text{moiré}}-k_{{\text{NbSe}}_{2}})$$

with $k_{\mathrm{Gr}}$ and $k_{{\text{NbSe}}_{2}}$ denoting the crystal momenta of electrons in the graphene and NbSe_2_ layers, respectively, and $G_{\text{moiré}}$ a reciprocal lattice vector of the moiré potential. In this framework, an electron in a state $|k_{\text{Gr}}\rangle$ can tunnel coherently into a state $|k_{{\text{NbSe}}_{2}}\rangle$ provided this condition is met.

For the first‐order moiré harmonics, this interlayer tunnelling gives rise to the arc‐like spectral features observed near the M points of the NbSe_2_ Brillouin zone, with the highest intensities found at the momentum‐space locations where the wavefunction overlap $\left\langle k_{\text{Gr}}|k_{{\text{NbSe}}_{2}}\right\rangle$ is maximal. This spectral weight distribution is entirely consistent with our experimental measurements, where we find the intensity of the replica bands to be strongly peaked where they overlap the Nb‐derived Fermi surfaces (see also Figure S7, Supporting Information). We note that, if the replica observed experimentally were due to simple final‐state effects in our measured photoemission spectra, six replicas would be expected surrounding the original Dirac states at the graphite K/K′ points, each with approximately the same spectral weight, and each exhibiting the same angle‐dependent intensity variations as for the measured primary graphite states at K_Gr_,^[^ ^48^ ^]^ but simply shifted in momentum. This is not what we observe here. As is typically observed due to the so‐called “dark corridor” effect,^[^ ^48^, ^49^, ^50^ ^]^ the spectral intensity of our primary graphite states is strongly peaked within the first graphite Brillouin zone (see also Figure S5, Supporting Information), almost the opposite k‐dependent variation to that observed of our replica features (see Figure S6, Supporting Information). Finally, additional moiré replicas which do not overlap the Nb‐derived K‐pockets (see also Figure S5a, Supporting Information) are much weaker than the one intersecting the Nb states. This directly indicates that the observed replica features derive from the initial‐state electronic structure, and reflect a moiré‐induced band hybridization between the graphite and the NbSe_2_‐derived states.

Our calculations indicate small but finite hybridization gaps open where the moiré replicas cross the primary NbSe_2_‐derived states (Figure 3c). This leads to momentum‐selective hybridization gaps forming in the Fermi surface, localized to the specific k‐points where the moiré replicas intersect the NbSe_2_‐derived states (Figure 2c). Such behavior is indicative of a coherent band anti‐crossing process, forming mini‐gaps governed by the momentum‐matching condition $k_{{\mathrm{NbSe}}_{2}}=k_{\mathrm{Gr}}+G_{\text{moiré}}$. This leads to a “rim”‐like gap structure, reminiscent of the hot spots encountered in CDW phases of transition metal dichalcogenides, including NbSe_2_,^[^ ^51^, ^52^ ^]^ where localized coupling leads to selective Fermi surface reconstruction. It may be captured within a minimal two‐level Hamiltonian of the form

(2) $$\hat{H}\left(k\right)=\left(\begin{array}{cc} \varepsilon_{{\mathrm{NbSe}}_{2}}\left(k\right) & t(k,k+G_{\text{moiré}})\\ t^{*}\left(k,k+G_{\text{moiré}}\right) & \varepsilon_{\mathrm{Gr}}\left(k+G_{\text{moiré}}\right) \end{array}\right)$$

from which the local hybridization gap follows as $\Delta_{\text{moiré}}(k)=2|t(k,k+G_{\text{moiré}})|$.

While it is difficult to directly resolve the gap in our measured dispersions due to the small associated energy scales, we note that the underlying Nb‐derived K‐barrel Fermi surfaces observed experimentally are more warped than would be expected for an isolated monolayer of NbSe_2_.^[^ ^31^ ^]^ In particular, the spin splitting almost vanishes along the M‐K direction. This is suggestive of the formation of momentum‐selective hybridization gaps by the moiré interaction, that open where the primary NbSe_2_ and graphite replica Fermi surfaces cross each other. This motivates future studies of aligned NbSe_2_/graphite samples by high‐resolution quasiparticle‐interference imaging, where the corresponding gap openings at the Fermi surface should be clearly resolvable.^[^ ^33^ ^]^ Interestingly, as shown in Figure 3c, our calculations reveal that a finite spin polarization should emerge in the graphite bands near the hybridization gaps. This phenomenon can be directly attributed to the off‐diagonal tunnelling elements in Equation (2), which mediate spin transfer from the strongly spin‐polarized NbSe_2_ states, $\varepsilon_{{\mathrm{NbSe}}_{2}}$, to the nominally spin‐degenerate states in graphite, $\varepsilon_{\mathrm{Gr}}$. The resulting spin texture in the graphite‐derived bands highlights that the moiré interaction can provide a route to proximity‐induced spin–orbit coupling; something that is being actively sought for spintronic functionality in graphene‐based heterostructures.^[^ ^53^, ^54^ ^]^ We note that a similar spin–orbit proximity effect would also be expected in heterostructures formed from mechanical exfoliation, and would be of interest to study, e.g., in transport devices of this system, as well as via direct spin‐resolved ARPES investigations of the electronic structure.

As well as these “first‐order” replicas of the primary graphite states, our calculations (Figure 2b,c) reveal a rich hierarchy of additional replica features. Near the Γ point, a rather flat feature is evident in the calculated band dispersions along the Γ‐K direction, close to the band bottom of the Nb‐derived conduction bands (Figure 2b). This results from a moiré‐induced replica of the primary NbSe_2_‐derived states (see also the Nb‐derived Γ‐ and K‐barrel replicas evident in our Fermi surface calculations, Figure 2c). Moreover, weak spectral weight from steep graphite‐like band dispersions is also visible centred around the Γ point (see right inset of Figure 2b). This can be assigned as second‐order moiré replicas of the graphite states (Figure 1d). At second and higher orders, the same selection rule as in Equation (1) applies, but the tunnelling amplitude is significantly diminished. The second‐order graphite replicas therefore only yield faint features in our calculations, e.g., near the Γ point. Nonetheless, while extremely weak, finite signatures of both of the above features can be observed in our experimental measurements (inset close to Γ in Figure 2a). While the graphite states are again slightly displaced in momentum from those in our calculations due to the enforced commensurability in the latter, they are otherwise in excellent qualitative agreement.

### 2.3 Impact on CDW Order

The resulting moiré heterostructure formation thus fundamentally reshapes the low‐energy electronic structure of this system. This can be expected to have a direct impact on the collective states which NbSe_2_ hosts. Indeed, recent scanning tunnelling microscopy quasiparticle interference measurements from rotated structural domains of NbSe_2_/graphene have shown evidence for momentum‐dependent modulations of the superconducting gap arising due to the substrate‐epilayer moiré lattice.^[^ ^33^ ^]^ While for a different azimuthal alignment in the measurements shown here, our direct observation of moiré replicas in the electronic structure of NbSe_2_/graphite heterostructures explicitly provides spectroscopic evidence of such moiré interactions and supports their importance for understanding superconductivity in ML‐NbSe_2_. This therefore motivates further studies, such as scanning tunnelling microscopy, optical and transport measurements, investigating the detailed dependence of superconductivity and moiré order on interlayer twist angle (see Figure S2c, Supporting Information), which calculations predict should lead to a rich response.^[^ ^55^, ^56^ ^]^

**Figure 4:**
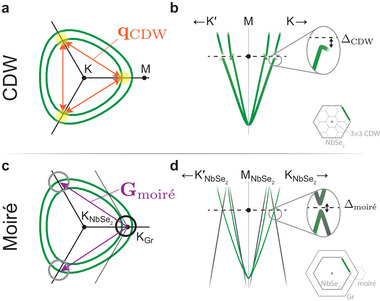
Impact of moiré replicas on the CDW order in NbSe_2_. a) Schematic K‐barrel Fermi surfaces of NbSe_2_, showing the wavevectors of the 3 × 3 CDW, $q_{\mathrm{CDW}}$ and b) the corresponding opening of CDW gaps at the Fermi level, with an energy scale $\Delta_{\mathrm{CDW}}\approx 4$ meV.^[^ ^51^ ^]^ c) Corresponding schematic K‐barrel Fermi surfaces for ML‐NbSe_2_/graphite heterostructures, showing in addition the primary graphite states (black circle) and their moiré replicas (grey circles), shifted by $G_{\text{moiré}}$. d) The intersection of the NbSe_2_ states and moiré replicas opens hybridization gaps (DFT‐estimated $\Delta_{\text{moiré}}\approx 10$ meV) at the same k‐space locations where the CDW gap is maximal (*cf*. (b)). The insets at the bottom right in (b) and (d) show the corresponding Brillouin zones, showing also the (3 × 3) CDW and moiré zones, respectively.

Moreover, we note that the (3 × 3) CDW instability in NbSe_2_ is known to open its dominant energy gaps at the corners of the K‐barrel Fermi surfaces (**Figure** **4**a,b), with the largest gap opening on the smaller pocket.^[^ ^51^ ^]^ Experimentally, we find that the graphite replicas cross the Nb‐derived Fermi pockets precisely at this momentum‐space location (Figure 3a; Figures S5 and S7, Supporting Information), as shown schematically in Figure 4c. We estimate the energy scale of the resulting moiré‐induced hybridization gaps at the Fermi level (Figure 4d) as $\Delta_{\text{moiré}}\approx 10$ meV from our full DFT supercell calculations. This is larger than the largest reported CDW gaps in the bulk,^[^ ^51^ ^]^ and so can be expected to directly suppress the thermally‐sensitive CDW instability. Indeed, such a competition may provide a microscopic mechanism to rationalise the controversial thickness‐dependent CDW evolution in NbSe_2_: For ML‐NbSe_2_/graphene heterostructures, where this competition can be expected, the CDW has been shown to become stable only at or below the bulk transition temperature,^[^ ^30^ ^]^ while it is reported that a graphene capping layer suppresses the CDW instability in few‐layer NbSe_2_.^[^ ^57^ ^]^ In contrast, for samples supported on insulating substrates, where there are no states available to open hybridization gaps at the Fermi level from moiré coupling at the CDW wavevector, the CDW is known to be significantly strengthened in the monolayer limit.^[^ ^34^, ^58^ ^]^ To further investigate this, we have performed structural optimization calculations to compare the CDW stability for an isolated monolayer of NbSe_2_ as compared to the NbSe_2_/graphene heterostructure. The optimization allowed relaxation of all Nb and Se atomic positions in a 6 × 6 supercell. For the isolated ML‐NbSe_2_, we obtained an energy difference of approximately 10 meV per formula unit between the CDW (distorted) and undistorted lattices. This suggests CDW phase stability up to about 100 K for the monolayer alone. In contrast, for the heterostructure geometry, the energy difference was negligible, ∼−0.1 meV per formula unit. This indicates that the enhanced CDW tendency of the isolated monolayer is strongly suppressed when NbSe_2_ is supported on a graphite‐like layer. Direct experimental electronic structure comparisons of ML‐NbSe_2_ on graphite vs. insulating substrates would therefore be of strong interest for future comparisons.

## 3 Conclusion 

Our work demonstrates how moiré interactions can readily be realized in MBE‐grown van der Waals heterostructures of transition‐metal dichalcognides. Despite the large lattice mismatch in our NbSe_2_/graphite system, we find that the moiré coupling is significant for the low energy physics of the system. In particular, our results establish that, beyond the well‐established ability of moiré lattice formation to stabilize a plethora of correlated states in 2D materials, it may also act to *destabilize* collective states that are inherent to the constituent materials. As many of the 2D materials which host such states are intrinsically air sensitive, we expect that our epitaxial moiré approach will provide a powerful materials platform for investigating this.

## 4 Experimental Section

### Molecular Beam Epitaxy

The samples were grown on bulk natural graphite substrates with thickness of several hundred microns, which were secured to Ta foil chips using high‐temperature carbon paste. Before growth, the paste was cured at approximately 300 °C, and the substrates were then degassed at 600 °C for approximately 2 hours at a pressure of ∼10^−7^ mbar. The graphite substrates were exfoliated just before being loaded into the vacuum system, where they were further degassed in the load‐lock at 200 °C for 10 hours. Immediately before the thin film growth, they were finally annealed at 800 °C for ∼30 minutes.

The NbSe_2_ films were grown at a nominal growth temperature of 700 °C, measured from a thermocouple behind the substrate. Nb was evaporated from a *FOCUS EFM* electron beam evaporator, maintaining a supply of 2.0 nA measured by the integrated flux monitor. Se was evaporated from a valved cracker cell with temperatures of 163 °C and 500 °C for the tank and cracking zone, respectively, resulting in a beam equivalent pressure of 2 × 10^−7^ mbar. A 3N5 pure Nb rod and 5N pure Se granules were used as source materials. The growth was monitored by reflection high‐energy electron diffraction, confirming a high monolayer coverage after the growth time of 2 h 10 min, as shown in Figure S1 (Supporting Information). The growth was ended by cutting off the Nb supply and the sample was cooled down under the Se pressure, which was cut off at 300 °C. The slow growth rates, enhanced nucleation resulting from excited ions from the Nb source,^[^ ^28^ ^]^ and high‐quality surfaces of the natural graphite substrates are all essential to yield high‐quality epitaxial layers here.

### Angle‐Resolved Photoemission Spectroscopy

Micro‐ARPES measurements were performed at the nano‐ARPES endstation of the I05 beamline at Diamond Light Source, using a capillary focusing optic with a beam spot size on the sample of 4–5 µm. The samples were transferred from the growth system to the beamline endstation using a vacuum suitcase. The samples were cooled to approximately 25 K and measured using a Scienta DA30 electron analyzer. The analyser slit direction is represented as the $k_{x}$ direction in the data. The photon energies and polarizations used are indicated in the figure captions. ARPES data analysis was performed using the *peaks* package.^[^ ^59^ ^]^

## 5 Computational Section

To construct a realistic model of the electronic structure, we first performed DFT calculations of a NbSe_2_/graphene heterostructure employing the Perdew–Burke–Ernzerhof exchange–correlation functional,^[^ ^60^ ^]^ as implemented in the VASP package.^[^ ^61^, ^62^ ^]^ The simulations were carried out on a trigonal supercell comprising 2 × 2 unit cells of NbSe_2_ and 3 × 3 unit cells of graphene. To eliminate lattice mismatch within the supercell, the in‐plane lattice constant of graphene was isotropically compressed from its experimental value of 2.460 Å^[^ ^63^ ^]^ to 2.294 Å, while the NbSe_2_ lattice constant was maintained at its experimental value of 3.442 Å.^[^ ^64^ ^]^ A vacuum layer of 18 Å was introduced along the crystallographic *c*‐axis to prevent spurious interactions between periodic images. The atomic positions were fully relaxed until the residual forces on all atoms fell below 0.001 eV/Å, using a kinetic energy cut‐off of 400 eV for the plane waves included in the basis set. Spin–orbit coupling was explicitly included, and the Brillouin zone was sampled using a 12 × 12 × 1 Monkhorst–Pack k‐mesh. From the converged band structure, we extracted the energy offset between the graphene Dirac point and the Fermi level.

We then performed analogous DFT calculations for isolated monolayers of NbSe_2_ and graphene, using the same unit cell geometries but without structural relaxation. The resulting Hamiltonians were projected onto Wannier functions^[^ ^65^ ^]^ using Nb‐4*d*, Se‐4*p*, and C‐2*p*_*z*_ orbitals as projection centres, yielding 88‐band (${\hat{H}}_{{\text{NbSe}}_{2}}$) and 18‐band (${\hat{H}}_{\text{Gr}}$) tight‐binding models, respectively. To align the energy scales of the two subsystems, appropriate onsite energy corrections were applied based on the energy shift determined in the full heterostructure calculation. The total Hamiltonian of the heterostructure was then constructed as $\hat{H}={\hat{H}}_{{\text{NbSe}}_{2}}+{\hat{H}}_{\text{Gr}}+{\hat{H}}_{\text{int}}$, where the interlayer coupling term ${\hat{H}}_{\text{int}}$ was derived by introducing orbital‐dependent hopping interactions between C and Nb atoms as well as C and Se atoms. Specifically, ${\hat{H}}_{\text{int}}$ includes coupling terms between all C‐2*p*_*z*_ orbitals and in‐plane $\left\{\text{Nb-}4d_{xy},\text{Nb-}4d_{x^{2}-y^{2}}\right\}$ and out‐of‐plane Nb‐$4d_{z^{2}}$ orbitals, as well as between all C‐2*p*_*z*_ orbitals and in‐plane {Se‐4*p*_*x*_, Se‐4*p*_*y*_} and out‐of‐plane Se‐4*p*_*z*_ orbitals of the Se layer adjacent to graphene. The interlayer interaction $t_{\mathrm{ij}}$ between *i* = C and *j* = {Nb, Se} was modeled as an exponentially decaying function $t_{ij}=\lambda_{j}\exp\left(-r_{ij}^{\left|\right|}/\xi \right)$, where $r_{\text{ij}}^{\left|\right|}$ is the in‐pane distance between sites *i* and *j*. This interlayer coupling term modulates the orbital overlaps computed from the Wannier basis, thereby filtering active tunnelling channels in the hybridization spectrum according to the orbital composition and symmetry of the constituent Bloch states. The values $\lambda_{\mathrm{Nb}}=0.30$ eV and $\lambda_{\mathrm{Se}}=0.36$ eV were chosen empirically so that the spectral weight projected onto the NbSe_2_ layer closely reproduces the interfacial patterns seen in ARPES—in particular, the folded replicas which are mixed into the top NbSe_2_ valence bands. For simplicity, the distance–decay prefactor is taken identical for in–plane and out‐of‐plane channels, with a fixed modulation length ξ=3 Å. Unfolded band structures and constant–energy maps were then computed using the procedure of ref. [66], enabling direct comparison with the experimental ARPES spectra.

## Conflict of Interest

The authors declare no conflict of interest.

## Supporting information

Supporting Information

## Data Availability

The data that support the findings of this study are openly available in University of St Andrews Research Portal at https://doi.org/10.17630/8c491d8c‐ec00‐422b‐93b1‐500827a2324a.^[^ ^67^ ^]^
