# Supplementary material for: Resonant Interlayer Coupling in NbSe2‐Graphite Epitaxial Moiré Superlattices
Source: Adv Mater. 2025 Dec 19;38(9):e11262. doi: 10.1002/adma.202511262 (PMC12902599; doi:10.1002/adma.202511262)
Supplement: Supplementary file 1 — Supporting Information [file ADMA-38-e11262-s001.pdf]

ADVANCED MATERIALS

Supporting Information

for *Adv. Mater.*, DOI 10.1002/adma.202511262

Resonant Interlayer Coupling in $NbSe_2$-Graphite Epitaxial Moiré Superlattices

Shu Mo, Ksenija Kovalenka, Sebastian Buchberger, Bruno K. Saika, Anugrah Azhar, Akhil Rajan, Andela Zivanovic, Yu-Chi Yao, Rodion V. Belosludov, Matthew D. Watson, M Saeed Bahramy and Phil D.C. King**

Supplementary Information: Resonant interlayer coupling in $NbSe_2$-graphite epitaxial moiré superlattices

S. Mo,[1,*] K. Kovalenka,[2,*] S. Buchberger,[1,3] B.K. Saika,[1] A. Azhar,[2,4] A. Rajan,[1] A. Zivanovic,[1] Y.-C. Yao,[1,3] R.V. Belosludov,[5] M.D. Watson,[6] M.S. Bahramy,[2,†] and P.D.C. King[1,‡]

[1]*SUPA, School of Physics and Astronomy, University of St Andrews, St Andrews KY16 9SS, United Kingdom*
[2]*Department of Physics and Astronomy, University of Manchester, Manchester M13 9PL, United Kingdom*
[3]*Max Planck Institute for Chemical Physics of Solids, Nöthnitzer Strasse 40, Dresden 01187, Germany*
[4]*Physics Study Program, Faculty of Science and Technology, Syarif Hidayatullah State Islamic University Jakarta, Tangerang Selatan 15412, Indonesia*
[5]*Institute for Materials Research, Tohoku University, Sendai 980-08577, Japan*
[6]*Diamond Light Source Ltd, Harwell Science and Innovation Campus, Didcot OX11 0DE, United Kingdom*

(Dated: October 21, 2025)

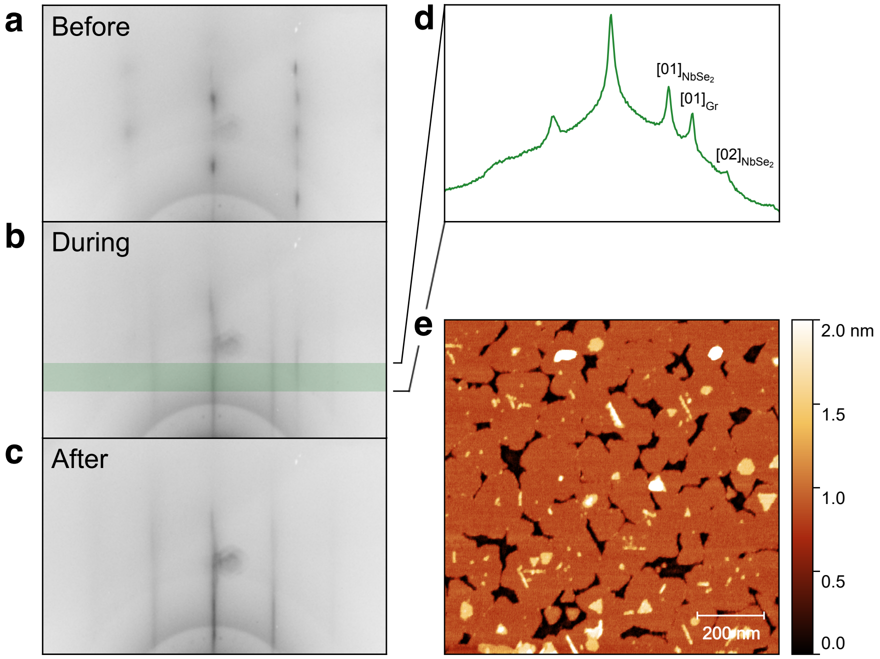

Supplementary Fig. 1. Materials characterisation. (a-c) Reflection high-energy electron diffraction measurements of an epitaxial $NbSe_2$/graphite heterostructure: (a) substrate pattern measured before the growth; (b) measurement 65 min into the growth with partial layer coverage, showing clear diffraction streaks from both the graphite and $NbSe_2$ layers; (c) at the end of the growth after 130 min, showing only the film streaks. (d) A line cut through the measured diffraction pattern shown in (b). From this, we extract a bulk-like lattice constant of 3.46 ± 0.05 Å of the $NbSe_2$ layer, indicating the absence of any detectable strain being coupled into the epilayer. (e) Corresponding atomic force microscopy measurements, indicating the growth of well-ordered $NbSe_2$ with near-complete coverage of a single monolayer and a percolative path. Similar measurements are obtained across the growth substrate.

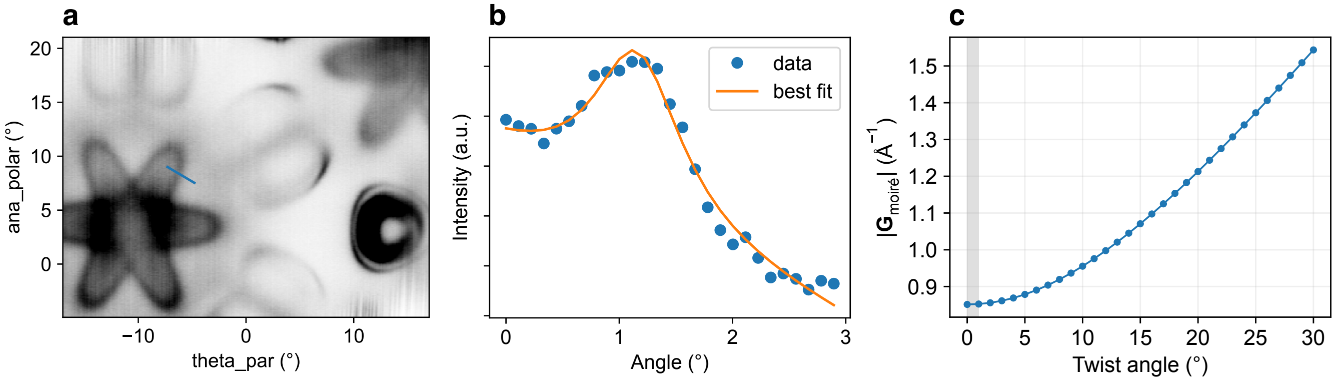

Supplementary Fig. 2. (a) A raw measured constant energy contour at $E - E_\mathrm{F} = -1$ eV in angle-space. The blue line indicates the range over which an angular distribtuion curve is extracted in (b), showing the intensity as a function of angle. A linear background and a Lorentzian line shape were used to fit the data, with a resulting full width at half maximum of $\lesssim 1°$. (c) Calculated values of the moiré vector as a function of the twist angle between ML-$NbSe_2$ and graphite. The grey shaded region shows that there is minimal variation expected in the moiré periodicity over the range of twist angles permitted by our angular analysis above.

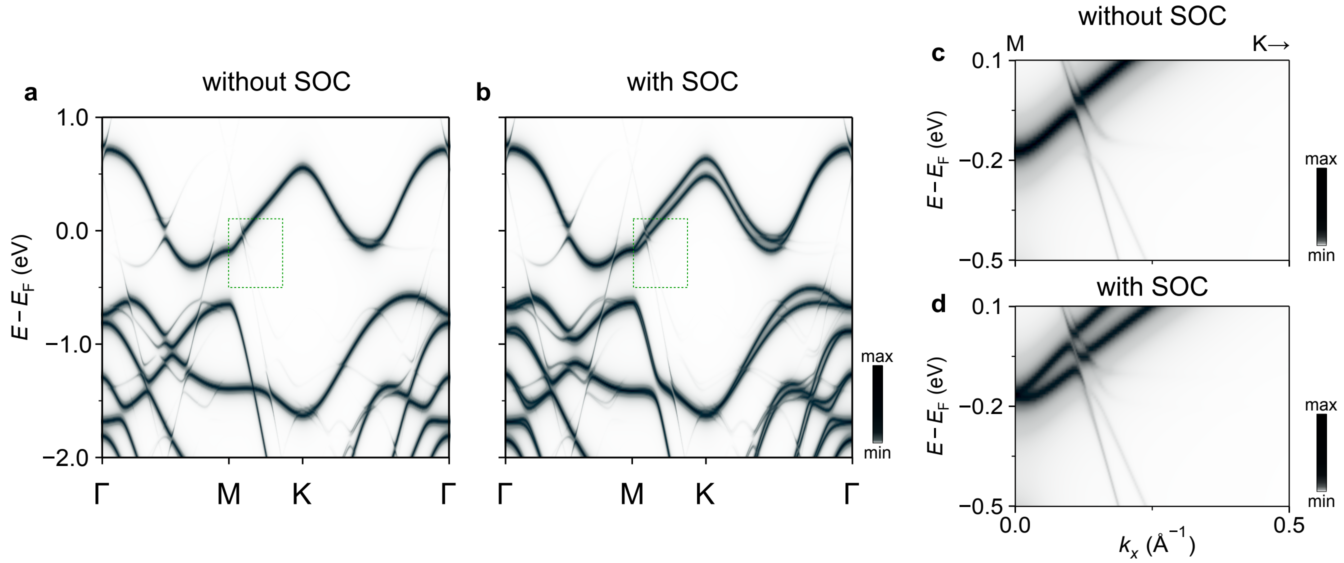

Supplementary Fig. 3. Influence of spin–orbit coupling on the electronic structure. Theoretical calculations of the ML-$NbSe_2$/graphene heterostructure bands along Γ-M-K-Γ (in the $NbSe_2$ Brillouin zone) (a) excluding and (b) including spin-orbit coupling. The development of a spin-splitting of the Nb-derived conduction band states is evident in the latter. (c,d) Magnified portion of the band structure along the M-K direction, showing the graphene resonance in the $NbSe_2$ bands (c) without and (d) with spin–orbit coupling.

* These authors contributed equally
† m.saeed.bahramy@manchester.ac.uk
‡ pdk6@st-andrews.ac.uk

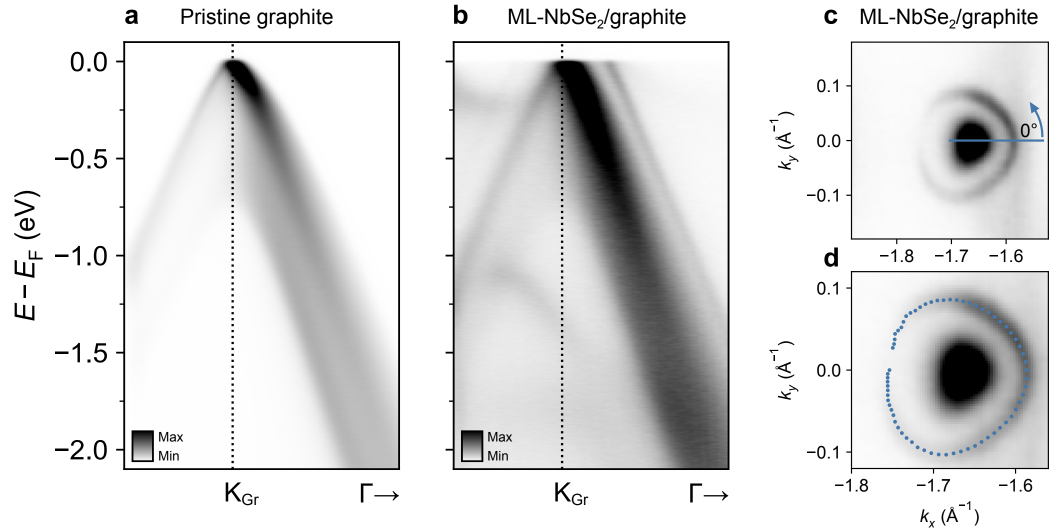

Supplementary Fig. 4. Interlayer charge transfer. ARPES measurements ($hv = 70$ eV, sum of CL and CR polarisations) of the dispersion along Γ-K in the vicinity of the graphite K point for (a) a pristine graphite sample, and (b) a $NbSe_2$/graphite heterostructure. The latter shows an additional linear band split-off from the k_z-dispersive bulk graphite states. This indicates charge transfer from the graphite to the $NbSe_2$ states, hole doping the former. (c) Corresponding Fermi surface measurements in the vicinity of the graphite K point show a well-defined trigonally-warped hole pocket derived from this split-off state. To extract the corresponding Luttinger density, we fit MDCs as a function of the azimuthal angle around the Fermi surface (see arrow). The resulting peak positions are shown in (d).

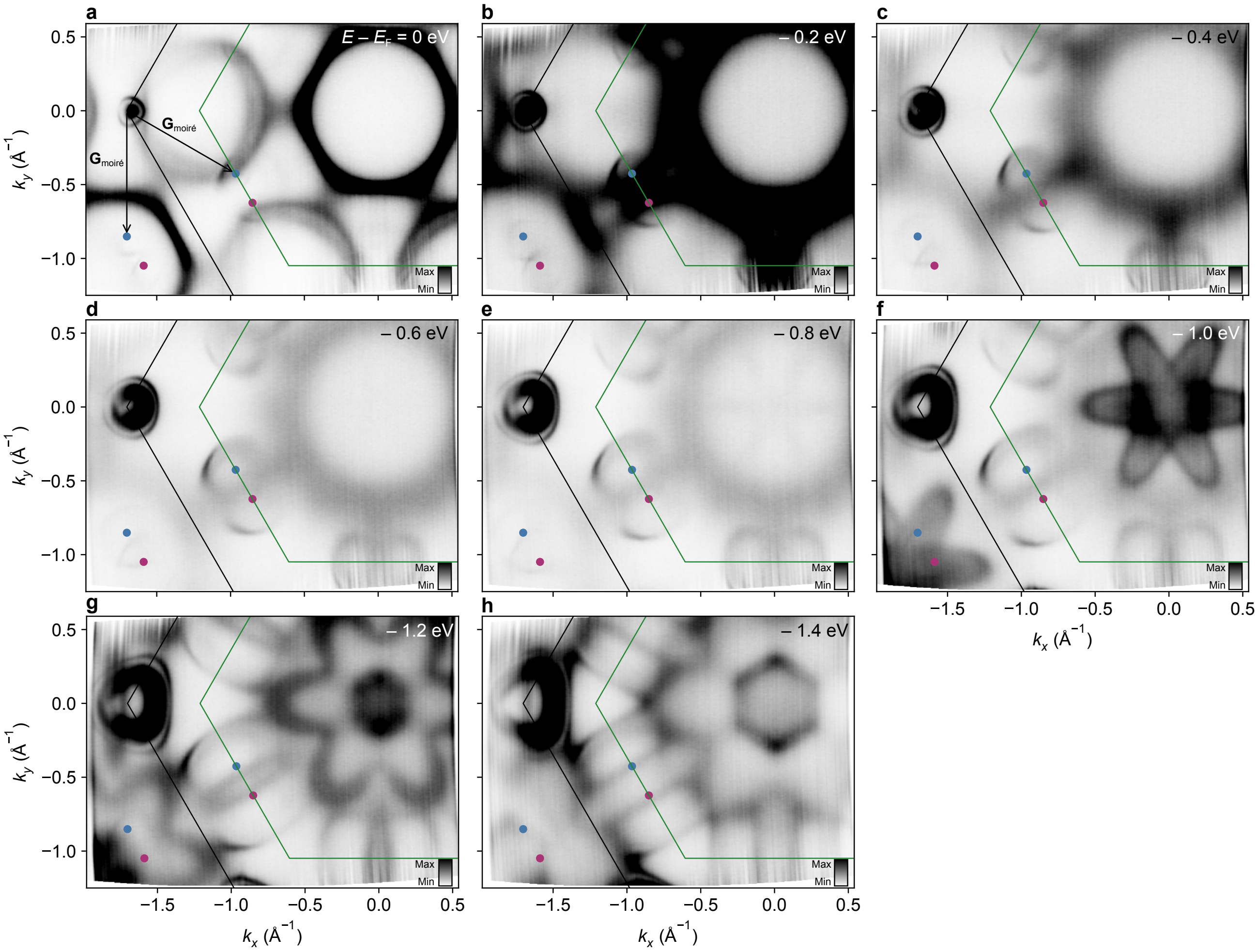

Supplementary Fig. 5. Constant energy contours. Measured (a) Fermi surface and (b-h) constant energy contours (see labels for energy). The measurements are the same as shown in Fig. 2(c) of the main text, but are shown here over an extended energy and momentum range and with enhanced contrast.

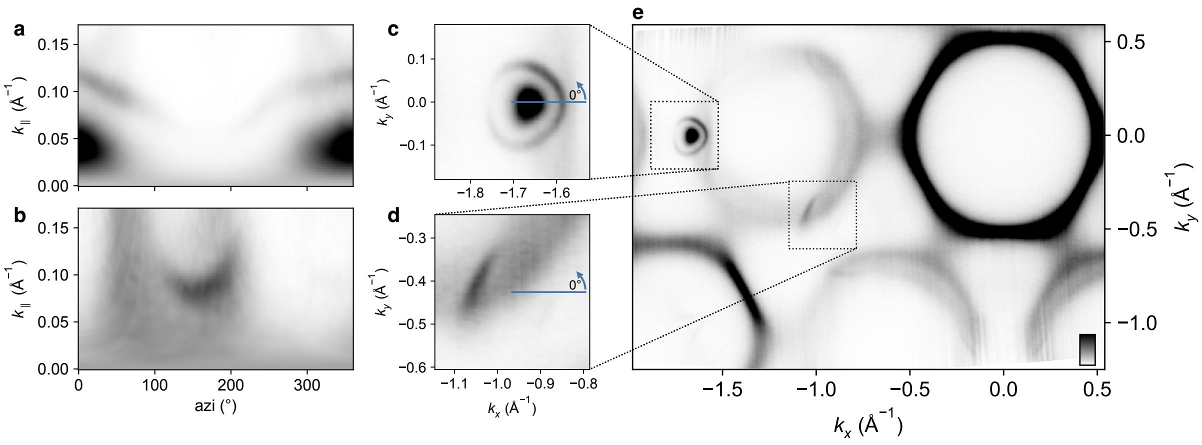

Supplementary Fig. 6. Comparison of the azimuth-dependent spectral weight variation between the primary graphite states and the moiré replica. (a) and (b) show the spectral weight as a function of the azimuthal angle for the primary graphite Fermi pocket and the associated moiré replica. (c) and (d) show how the azimuthal analysis was extracted from (e) the measured Fermi surface. The two centres in (c) and (d) were determined from the calculated high-symmetry points and moiré vector.

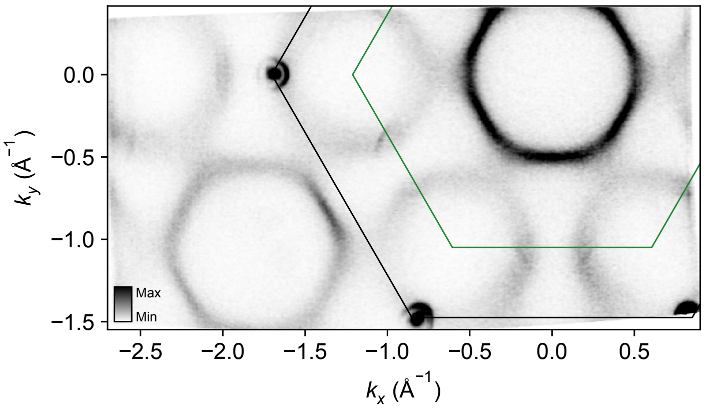

Supplementary Fig. 7. Additional observation of graphite moiré replicas. Additional ARPES Fermi surface measurements performed at a photon energy of $hv = 127$ eV, presented as the sum of spectra measured in CL and CR polarisations, showing clear spectral weight of the graphite moiré replicas where they cross the Nb-derived conduction band states.

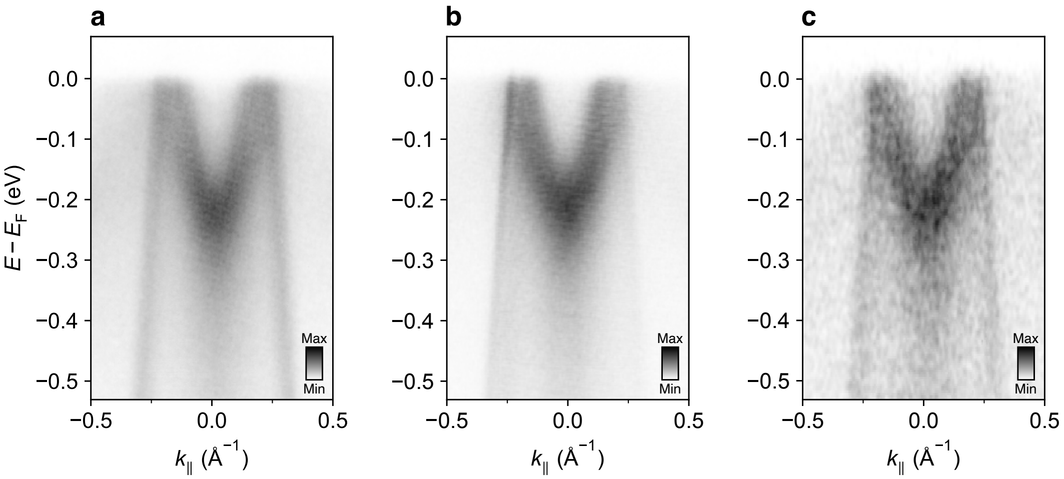

Supplementary Fig. 8. Additional observation of graphite moiré replicas. Additional measured dispersions along K-M-K$'$ at (a) 55 eV (in CL polarisation), (b) 70 eV (sum of spectra measured in CL and CR polarisations), and (c) 127 eV (sum of spectra measured in CL and CR polarisations), extracted from Fermi surface maps, confirming the presence of the graphite moiré replicas at various photon energies.
